# How, when, and for whom: decisions regarding remote neuropsychological assessment during the 2020 COVID-19 pandemic

**DOI:** 10.1186/s13584-021-00465-x

**Published:** 2021-05-03

**Authors:** Ayala Bloch, Sari Maril, Gitit Kavé

**Affiliations:** 1 Department of Psychology, Ariel University, 65 Ramat HaGolan St, Ariel, Israel; 2 The National Institute of Neuropsychological Rehabilitation, Tel Aviv, Israel; 3 Department of Education and Psychology, The Open University, Ra’anana, Israel; 4 Center for Memory and Attention Disorders, Sourasky Medical Center, Tel Aviv, Israel

**Keywords:** Neuropsychology, Neuropsychological assessment, Tele-neuropsychology, COVID-19

## Abstract

Neuropsychological assessment provides crucial information about cognitive, behavioral, and socioemotional functioning in medical, educational, legal, and social contexts. During the 2020 COVID-19 pandemic, the Israeli Ministry of Health initially mandated that all psychological assessments be postponed. However, as referrals to time-sensitive, high-need, and high-stakes assessments began to accumulate, it became necessary to consider remote solutions. In the current paper, we describe the considerations that affected the transition to remote activity in a prominent Israeli provider of neuropsychological assessment and rehabilitation services, referring to technological and environmental conditions, cognitive requirements, and tasks, as well as to legal, regulatory, and funding issues. After discussing how assessments should be conducted to maximize feasibility and validity while minimizing risks to clients and clinicians, we propose a preliminary model for deciding whether specific referrals warrant remote administration. The model delineates key factors in decisions regarding remote assessment, emphasizing the distinct roles of the referring clinician and the neuropsychologist who conducts the assessment, and highlighting the need for collaboration between them. The abrupt need for remote assessments during the pandemic required a quick response with little preparation. The lessons learned from this process can be applied in the future, so that the need for remote services can be met with greater certainty and uniformity.

## Background and aims

Over a year into the 2020 COVID-19 pandemic, decisions about the provision of remote health services are still largely in the hands of individual clinicians and medical service providers. Given the resulting uncertainty and inconsistency, the Israeli healthcare system would clearly benefit from well-defined policies to support decisions concerning remote services. Though many of the issues encountered in defining such policies depend on the specific field and service in question, some are shared by several fields.

In the current paper, we discuss the provision of remote neuropsychological assessment during the pandemic, propose key guidelines for determining whether or not to conduct such assessment, and consider the applicability of these guidelines to policy governing remote assessment services in Israel.

## Neuropsychological assessment

Neuropsychological assessment evaluates cognitive, behavioral, and socioemotional functioning, in relation to known or suspected neural damage resulting from injury or disease [1, 2]. Within health systems, it is primarily used to support medical diagnosis, guide treatment and rehabilitation plans, and predict functional potential and recovery [3]. It is also employed in a range of forensic and welfare-related contexts [4], for example in determining fitness to stand trial, vocational prognosis, or disability levels for medico-legal purposes [5].

Though methods and approaches vary, neuropsychological assessment generally involves some or all of the following components: an in-depth interview, review of relevant medical history documents and caregiver accounts, a battery of standardized tests evaluating specific cognitive abilities, and questionnaires addressing functional, behavioral, and socioemotional factors [6]. Although there are some computerized assessment tools [7], most assessments involve face-to-face interaction and test administration, and rely heavily on information gained by the neuropsychologist through observing and communicating with the client. The length and content of each assessment are based on its specific objective, or referral question. To illustrate, an assessment conducted to guide a comprehensive rehabilitation plan for a patient with traumatic brain injury would employ a larger and more diverse set of tools than a focused assessment aiming to shed light on the differential diagnosis of an elderly patient reporting memory deficits.

In Israel, neuropsychological assessments are conducted by certified psychologists at hospitals and community-based clinics [8]. They are included in the medical services package and, depending on the objective and the referring organization, can be funded by health plans, the National Insurance Institute, the Ministry of Defense, the Ministry of Labor, Social Affairs and Social Services, insurance companies, or privately. In most cases, the referring clinician (e.g., physician, social worker), who often represents a funding organization, defines the question or issue to be addressed. Based on the referral question, the psychologist who conducts the assessment delineates its scope and nature. Policy regarding neuropsychological assessment in Israel, alongside other types of psychological assessment, falls under the authority of the Ministry of Health Council of Psychology and is regulated by the office of the National Psychologist.

## Initial implications of COVID-19

In responding to the novel coronavirus pandemic, the Israeli Ministry of Health initially mandated that all psychological assessments be postponed to a later date, in accordance with national guidelines limiting contact between individuals in the workplace. The National Psychologist instructed clinics, medical centers, and independent clinicians offering neuropsychological services to discontinue face-to-face assessments, with no specific reference made to remote assessment. The wide-ranging, substantial, and potentially long-lasting psychological impact of the pandemic [9] was already becoming clear. However, as neuropsychological assessments specifically address known or suspected neural damage, no drastic rise in referrals was documented. Even at the normal rate, however, they began to accumulate, and it became clear that time-sensitive, high-need, and high-stakes assessments could not simply be put aside.

Similar conclusions were reached worldwide, causing organizations such as the American Psychological Association (APA) and the International Neuropsychological Society (INS) to initiate public discussions regarding the provision of remote psychological services in general, and neuropsychological assessments in particular. While many of the considerations and challenges associated with remote neuropsychological assessment are relevant to telehealth in general and telepsychology in particular, the aforementioned organizations noted that neuropsychological assessment is disproportionately burdened by pandemic constraints, because it relies on procedures that require in-person interaction, such as manipulation of physical materials, standardized interactions between assessor and client, and clinical observation [10]. In this sense, neuropsychological assessment differs from psychotherapy (including neuropsychological therapy), which is a more prevalent and increasingly popular application of telehealth with widely available guidelines and best practices [11, 12].

In Israel, the restrictions put in place during the initial lockdown were lifted in May 2020, at which point limited face-to-face assessments were possible under some circumstances, provided that certain guidelines (mandated for all psychology sessions) were followed. However, remote or hybrid assessments continued to be necessary in some cases due to pandemic-related issues such as individual quarantines, patient anxiety, and other clinical considerations.

## Pre-COVID-19 literature on remote neuropsychological assessment

Before the 2020 COVID-19 pandemic, the literature on remote neuropsychological assessment (also known as tele-neuropsychology or tele-assessment) largely addressed its use in response to geographic barriers, which are common in the United States and other large countries. Several studies have reported that neuropsychological assessments administered via video teleconferencing show good agreement with traditional in-person assessments [13–16], particularly for tasks that involve only verbal responses [17].

Some of the tele-neuropsychology solutions have used computerized tools, such as RBANS [18] and CANTAB [19], to evaluate cognitive decline in older adults [20]. In other studies, neuropsychologists at major medical centers have assessed clients at outlying clinics via video teleconferencing, moderated locally by non-specialist staff members [21, 22]. In this case, assessment tools are available at the local clinic, while the neuropsychologist provides remote instructions and supervision.

## Remote testing during COVID-19: recommendations and considerations

The aforementioned body of work has been cited to justify the provision of remote neuropsychological assessment, both before and during the COVID-19 pandemic. However, the solutions described are not necessarily sufficient under the current circumstances, as computerized assessments frequently do not provide the breadth of information needed to draw comprehensive conclusions and recommendations, and social and physical distancing requirements can preclude moderated assessments. Accordingly, prominent organizations and researchers worldwide have acknowledged the need to develop practice recommendations and guidelines for conducting remote neuropsychological assessment specifically in light of COVID-19 restrictions [23–26].

In this context, a comprehensive document was released by a workgroup of the Inter Organizational Practice Committee (IOPC), a committee of several key American organizations (e.g., Division 40 of the American Psychological Association, the American Board of Professional Neuropsychology) that coordinates advocacy and growth efforts in the field of neuropsychology [27]. This document cites relevant resources, establishes guidelines for telepsychology, and offers recommendations about how these practices might be extended specifically to tele-neuropsychology. Mirroring much of the content discussed in a webinar hosted by the International Neurological Society [28], the IOPC document summarizes several key issues to be considered before conducting remote neuropsychological assessment.

Some of these considerations involve the technological and environmental conditions required for remote administration [23, 25, 27, 28]. The neuropsychologist who conducts the assessment must have an appropriate video teleconferencing platform as well as relevant tasks. The client must have a computer, camera, and Internet connection that can support the required platforms, as well as a sufficiently quiet testing environment. Equally important, the client must be cognitively and physically capable of operating the required instruments or have a caregiver available to help. These conditions are not trivial in a significant percentage of cases referred to neuropsychological assessment, who often have notable cognitive deficits. Several authors and organizations have recently published broad specifications regarding available technological platforms for neuropsychological assessment, and addressed various privacy and cybersecurity considerations (e.g., [26, 29]).

An additional set of considerations involves the tests or tasks to be used remotely [24, 28]. Many tasks are not suitable for remote administration, for example because they rely on special equipment that is not available to clients in their homes. Other tasks, as well as questionnaires and interview techniques, can be adapted with varying degrees of ease and accuracy. However, given that normative data is generally collected under face-to-face conditions, norms are not valid under remote administration conditions [26]. Before the COVID-19 pandemic, Brearly et al. [17] conducted a meta-analysis of studies comparing remote neuropsychological testing to in-person testing methods. They found that verbally administered tests, including list learning, digit span, and verbal fluency tasks, were generally not affected by administration method, while remote versions of visual and motor tests like picture naming and clock drawing received less support. More recently, Barcellos et al. [30] reported further evidence that tests of verbal memory and processing speed could be effectively administered remotely.

Remotely conducted interviews do not afford neuropsychologists the opportunity to observe and connect with their clients as closely as their face-to-face counterparts. This is critical, because such observation, for example of behavior and facial expressions, is the basis for assessing cognitive, social, behavioral, and emotional functioning. Furthermore, the rapport, or alliance, between the practitioner and the client affects important variables such as trust, comprehension, motivation, and the working relationship [31], which in turn impact the quality of assessment results.

Beyond this, for tests that are copyrighted or otherwise protected by purchase terms, modification and transfer to clients at other locations can raise legal concerns. While legal issues will likely be solved with time, test selection is currently among the primary challenges that neuropsychologists face when conducting remote assessments. Still, depending on the specific question or objective, it is often possible to find a combination of tasks and methods that can enable reasonable assessment.

In addition to the technical issues related to administration and interpretation of remote neuropsychological assessments, a range of other legal, regulatory, and funding considerations must be addressed. Questions arise, for example, regarding which neuropsychologists are qualified to administer remote assessments, and whether they require special training or licensing [27]. In terms of regulation, while all neuropsychological assessments are subject to laws regarding medical ethics, privacy, and informed consent, these must be extended and adapted to address the specific risks of modified, remotely administered assessments. Clients must be informed, for example, of the increased privacy risks associated with electronic information transfer (e.g., video teleconferencing, cloud transfer, and storage of questionnaires), and the potentially decreased validity and reliability of modified assessment tools. Finally, the costs of assessment under remote conditions must be considered, as well as related funding and reimbursement policies.

Although neuropsychological assessment is provided through the Israeli health system [32], there were no remote services in the country before the current pandemic. As such, the challenges summarized above emerged for the first time shortly after the pandemic began. In the following section, we describe the decisions and actions taken by one organization in response to these challenges.

## Case illustration: response of an Israeli assessment and rehabilitation organization

The considerations and decisions associated with remote assessment during the COVID-19 pandemic are well-illustrated by the response of the National Institute of Neuropsychological Rehabilitation (henceforth, the Institute; a public non-profit organization), Israel’s largest provider of community-based neuropsychological rehabilitation services for individuals with acquired brain damage. Alongside rehabilitation interventions, neuropsychological assessment is one of the Institute’s primary areas of expertise, with close to 200 assessments conducted each year under normal circumstances.

When COVID-19 began to spread in Israel and the Ministry of Health ordered a nationwide lockdown, the Institute, like others worldwide [33, 34], immediately began transitioning to remote provision of all rehabilitation interventions. In the first few days of preparation, the Institute acquired and arranged the necessary video-teleconferencing equipment and infrastructure, mapped the needs and facilities of its clients and clinical staff, and provided staff members with a short remote therapy training program. Program heads were appointed for the two primary intervention components and a new set of client safety and privacy guidelines was produced for video-based therapy.

During the first weeks of the pandemic, while interventions were administered remotely, all assessment activities were halted. Recently published surveys now show that this was in line with a general trend, as neuropsychologists reported reluctance or inability to deliver neuropsychological assessments when the pandemic began [35, 36]. However, as the pandemic continued and referrals began to accumulate, preparations began for remote assessment. A team of clinicians reviewed the relevant literature and assembled a new assessment battery with a greater number of computerized tests and fewer non-verbal tests than normally administered. The team formulated a detailed administration protocol, converted questionnaires to electronic forms, and trained all other staff members. The decision to conduct remote assessment was granted on a per-client basis, following a comprehensive review of needs, resources, and assessment goals. Staff psychologists created a new electronic consent form with professional legal guidance to address the validity of remote assessment and inform clients that their computers would be accessed during the assessment.

During the first of Israel’s three official lockdown periods (March 25 to May 3, 2020), the Institute conducted 16 fully remote focused assessments. Twenty-seven additional assessments, defined as hybrid, were initiated during the lockdown period and continued with face-to-face sessions when restrictions were relaxed. In parallel, tens of referrals were deferred until restrictions eased and conducted face-to-face (with mandated precautions such as physical distancing and mask-wearing). The process and considerations guiding the decision to undertake or defer these assessments, including specific examples, are discussed below. In all cases, the decisions were made in collaboration with the referring clinician as well as with the client. In some cases, the neuropsychologist delivered a laptop computer to the client’s home, as well as stimuli, and paper and pencils for reproduction and drawing tasks. Some of the clients required more caregiver support than others to complete their assessments.

Retrospective observation of the tasks administered remotely during fully or partially remote assessments begun during the lockdown reveals that many were verbal in nature, in accordance with previous literature [17, 24, 30]. These included, for example, the Rey Auditory Verbal Learning Test (RAVLT, [37]), story recall [38], verbal fluency, reading, sentence completion, and several verbal intelligence subtests from the Wechsler Adult Intelligence Scale (WAIS [39];). Beyond this, they included self-report questionnaires, such as the Behavior Rating Inventory of Executive Function (BRIEF [40];) and the Beck Depression Inventory (BDI [41];), and a computerized general assessment battery. Meanwhile, the following measures were not assessed remotely, either because they were unnecessary in fully remote assessments or because face-to-face sessions became an option (hybrid assessments): performance tests (e.g., reproduction tasks), attention tasks (e.g., continuous performance), malingering tasks, and executive functioning tasks (e.g., card sorting).

Since limitations on contact in clinical settings have been relaxed, the Institute has continued to provide hybrid assessments, with both remote and face-to-face components, based on the needs and preferences of the client. Face-to-face meetings are currently conducted in accordance with all Ministry of Health guidelines. Alongside the transition to hybrid methods, the Institute continues to improve its remote assessment capabilities.

## Deciding who should be remotely assessed

The brief description of the Institute’s transition primarily illustrates considerations related to *how* remote neuropsychological assessment should be conducted, to maximize feasibility and validity while minimizing risks to clients. Another outcome of this process was the realization that decisions about *when* to conduct remote assessment require in-depth considerations. Before Ministry of Health guidelines allowed hybrid or face-to-face assessments, when remote assessment was the only available option, the Institute had to quickly determine which referrals could not be postponed until restrictions were lifted. It immediately became clear that both referring clinicians and assessment providers would benefit from collaborative decision-making guidelines.

The uncertain circumstances that prevailed during the first months of the pandemic did not initially allow for the development of such guidelines. However, following retrospective examination of the decisions made and a review of relevant literature, we can now propose a preliminary model that delineates the factors and processes involved in deciding whether specific referrals warrant remote or hybrid neuropsychological assessment.

Broadly, this model posits that the decision to assess a client remotely should be based on the interplay between five factors: (1) necessity of assessment; (2) urgency of referral question; (3) feasibility, in terms of technological limits or clinical contraindications that might preclude remote assessment; (4) confidence level, or the extent to which the neuropsychologist is confident that the assessment will provide accurate information under remote administration conditions; and (5) required resources and costs associated with remote assessment.

**Fig. 1:**
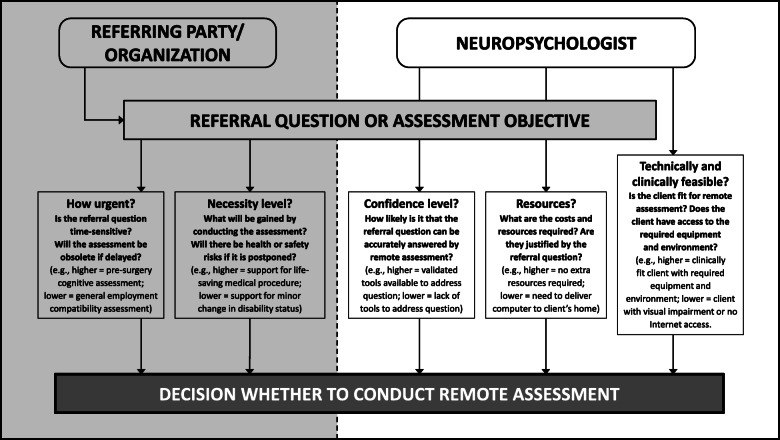
A decision-making model for remote neuropsychological assessment

As illustrated in Fig. 1, when an assessment is under consideration for remote or partially remote administration, some of the five factors are determined by the referring clinician, whereas other factors require the clinical judgement of a neuropsychologist. The five factors are discussed in greater detail below, alongside case examples from among tens of referrals for neuropsychological assessment received by the Institute during Israel’s first COVID-19 lockdown (March–May 2020).

### Factors determined by the referring clinician

As the one responsible for defining the objective of an assessment, or the question that the assessment would aim to answer, the referring clinician determines the first two factors in the model: necessity and urgency. Essentially, the referral question delineates the extent to which the assessment results are necessary to ensure the safety and wellbeing of the client (or others in the client’s environment), be it in a medical, social, or legal context, and how detrimental it would be to withhold or delay the assessment. To illustrate, a referral requesting a neuropsychological profile ahead of imminent surgery for epilepsy would be considered both necessary and urgent, as its results would be used to guide immediate medical actions and any delay in those actions would endanger the client. Meanwhile, a referral requesting elucidation of differential diagnosis between traumatic brain injury and post-traumatic stress disorder in a military veteran applying for financial benefits would be considered necessary but less urgent.

At the Institute specifically, which is not directly associated with a broader medical center, only a small percentage of referrals aim to guide immediate medical actions, such that most cases could be considered less urgent and necessary in terms of immediate safety. However, we often consider referral questions regarding suitability for treatment or social benefits highly necessary for ensuring client well-being. In accordance, we often determine urgency based on the timing of treatment programs or legal proceedings. Of tens of referrals received at the Institute during the first lockdown, all were labelled as moderately to highly necessary. Urgency varied more, and was often a deciding factor in whether or not to begin assessment remotely when the other factors did not preclude it.

### Factors determined by a neuropsychologist

In defining the necessity and urgency of the referral question, the referring clinician provides the neuropsychologist with the basis for evaluating the remaining three factors: feasibility, level of confidence, and required resources.

Technical and clinical feasibility is perhaps the most straightforward to determine, as the neuropsychologist should base the decision on whether the client can meet well-defined criteria for undergoing assessment remotely. Thus, regardless of necessity or urgency, a client whose home has no Internet connection or a client with insufficient cognitive capacity to independently operate a computer would not be able to undergo remote assessment. Clinical considerations can also affect feasibility; for example, a client who cannot use a computer due to a visual impairment, or due to anxiety related to technology use, would not be able to undergo remote assessment. Feasibility can therefore be considered a “go/no-go” factor: if an assessment is not deemed feasible, the other factors become irrelevant. The Institute indeed had to defer a small number of referrals during the first lockdown due predominantly to feasibility issues. Examples included a client who lived in a remote village with no Internet access and one whose general cognitive capacity was not high enough to enable remote assessment.

The neuropsychologist’s level of confidence in the results that can be obtained through remote assessment, given the referral question at hand, will be based largely on available tools. For example, as noted above, questions about verbal functioning are generally considered easier to answer using remote tasks, compared to questions about visuospatial abilities. In other cases, the clinician is confident that the referral question can be answered accurately based solely on tests that were specifically developed or validated for remote use. Another consideration in this category is expected reliance on face-to-face observation, which varies among assessments. Though not as clear cut as feasibility, confidence level can also be considered a primary deciding factor: it would not be advisable, for example, to address even an extremely urgent referral question through remote assessment if we did not expect it to provide a reasonably accurate answer. Of the referrals received by the Institute during the first lockdown, many of those that were undertaken either fully or partially remotely were aimed at evaluating compatibility with inclusion criteria for a specific treatment program, in a short, structured assessment comprising of computerized tasks and questionnaires that were relatively easy to adjust to remote conditions. Similar processes have been described in recent literature (e.g., [33]). In contrast, a referral that would need to rely largely on tests of medical malingering was deferred, as confidence in the results of such tests, when administered remotely, was very low.

In general, assessments aimed at guiding treatment and rehabilitation plans, or at recommending an appropriate care program or living arrangement, can be effectively administered remotely. Medical objectives, such as differential diagnosis or pre-surgery cognitive assessment, are associated with more moderate confidence levels, as are occupational questions. Forensic and legal questions, such as the ability to stand trial, and referrals related to determination of disability benefits may be more difficult to answer through remote assessment. However, these are general guidelines that have not been empirically examined.

The resources that the neuropsychologist must invest in preparation for a remote assessment can also influence whether it is conducted. Considerations in this realm might include extra time required for assessing a client remotely, as compared to face-to-face conditions, or the need to purchase additional tasks or equipment. A service that requires few resources can be relevant even for non-urgent referrals, but if it requires significant resources it may be better to conduct it at a later date, in the clinic rather than remotely. Indeed, of several referrals to assess clients who required computers to be delivered to their homes during the lockdown, the Institute was able to take on only some, due to limited resources. In these cases, the other four factors determined which assessments would take precedence.

### Allocation of responsibility and reliance on collaboration

Beyond delineating five key factors in decisions regarding remote assessment, the presented model emphasizes the distinct roles of the referring clinician and the neuropsychologist, as well as the resultant need for collaboration between them. Optimal decisions about whether to conduct a remote assessment during the COVID-19 pandemic or under other circumstances limiting face-to-face contact should rely on integrated knowledge from both sources. Any future policy guiding funding or approval for remote assessment should take this point into consideration.

### Deciding between assessment methods

While COVID-19 restrictions indeed continue to encourage preference for remote medical interactions to the extent possible, additional options like hybrid assessment have opened up. The considerations delineated by the model are relevant to deciding between different assessment methods. In this case, an additional factor – the risk involved in face-to-face assessment – should be considered as well. This is emphasized in an additional document made available by the IOPC [42], which reviews various options for conducting neuropsychological assessment and discusses considerations in choosing between them, based on associated benefits and potential risks.

## Conclusions and implications for policy

This paper presents the considerations found relevant by a prominent Israeli provider of neuropsychological assessment services following a rapid and unprecedented transition to remote activity in response to the COVID-19 pandemic. The transition elucidated the need for well-defined policies concerning the provision and nature of remote psychological assessment in general, and neuropsychological assessment specifically. Here, we recommend that the currently presented considerations and decision-making model serve as a basis for such policy in Israel, and support the Council of Psychology in formulating guidelines for clinicians involved in referral, assessment, and funding of neuropsychological assessment. Such guidelines should specifically address the factors to be considered in the decision to conduct an assessment, establish the required conditions, and delineate the roles of the involved professionals.

We believe that the Council of Psychology should formulate accepted guidelines that would help all relevant parties make decisions regarding remote assessments. In particular, we recommend that funders appoint a head neuropsychologist, who will be responsible for evaluating the necessity and feasibility of assessment given a particular objective or question. We also highlight the need to support and fund research and policy aimed at developing and validating measures and batteries specifically geared toward remote neuropsychological assessment, both by adjusting existing tests and by creating new ones. This relates to the more general understanding that the availability of such testing options, when the need for them arises, will enable better planning and execution of remote assessments. We also note, in this context, that alongside associated challenges, remote assessments have various advantages, among them convenience for clients and practitioners, flexibility, and accessibility to clients with social anxiety or impairments that limit their ability to leave their homes. In accordance, recent work shows high patient satisfaction ratings for remote neuropsychological assessment [43].

In conclusion, we note that the factors defined in the proposed decision-making model are in many ways generalizable to other limitations on the provision of neuropsychological assessment, less dramatic than a global pandemic. As such, while this decision-making model was developed in response to an urgent need to address remote assessment, its constituent factors could be the basis for a more general model. Furthermore, it stands to reason that these factors, and particularly the emphasis on integrative input from both the referring clinician and the neuropsychologist, are applicable to other psychological services requiring remote administration during the pandemic.

## Data Availability

Not applicable.
